# Evaluation of the knowledge, perception, and practice of nurses regarding sarcopenia: a descriptive study

**DOI:** 10.1186/s12877-025-06910-9

**Published:** 2026-01-05

**Authors:** Murad H. Taani, Heather Skuble, Charles Antwi-Boasiako, Dawn M. Wangler, Christopher J. Kerby, Mariam M. Kawafha, Suzan AlAbidi, Shaherah Yousef Andargeery

**Affiliations:** 1 School of Nursing, College of Health Professions and Sciences, University of Wisconsin-Milwaukee, 1921 E. Hartford Ave, P.O. Box 413, Milwaukee, WI 53211 USA; 2 Rush University College of Nursing, 600 S. Paulina, AAC 1080, Chicago, IL 60612 USA; 3 Instructional Academic Staff, UW-Oshkosh, Oshkosh, WI USA; 4 Faculty of Nursing, Yarmouk University, P. O. Box: 566, Irbid, Zip Code: 2116 Jordan; 5 College of Education, Humanities and Social Science, Al Ain University, Abu Dhabi, UAE; 6 Department of Nursing Management and Education, College of Nursing, Princess Nourah bint Abdulrahman University, P.O. Box 84428, Riyadh, 11671 Saudi Arabia

**Keywords:** Sarcopenia, Nursing, Muscle strength, Awareness, Older adults

## Abstract

**Background:**

Sarcopenia contributes to functional decline and adverse outcomes in older adults, yet little is known about nurses’ knowledge and practices regarding this condition. This study described nurses’ self-perceived knowledge, perceptions, and practices related to sarcopenia.

**Method:**

A descriptive, online survey was used to assess 109 nurses regarding sarcopenia.

**Results:**

Over half of the nurses reported limited knowledge of sarcopenia and were unsure whether it is preventable; 15.6% believed it was not preventable. Most had not received recent sarcopenia education (94.5%), were unfamiliar with diagnostic criteria (95.4%), and did not use objective assessment tools (96.3%). Nearly half overestimated the age at which muscle mass and strength begin to decline, and only 4.6% correctly identified sex-specific grip strength cut-offs. Confusion with frailty criteria was common (23.8%).

**Conclusion:**

Findings reveal substantial gaps in nurses’ knowledge and practice regarding sarcopenia in older adults. Enhanced education, clinical guidelines, and standardized assessment processes are needed to support timely recognition and management.

## Background

Sarcopenia is an important health concern, particularly among the aging population, marked by the gradual decline in skeletal muscle mass, strength, and function [3, 6, 9]. The European Working Group on Sarcopenia in Older People (EWGSOP2), a panel of international experts, developed the updated consensus definition and diagnostic criteria for sarcopenia. The EWGSOP2 emphasizes that low muscle strength is the primary parameter of sarcopenia, and when low muscle strength is present, sarcopenia is considered probable. A diagnosis is confirmed when low muscle quantity (i.e., muscle mass) is also present. When low muscle strength, low muscle mass, and poor muscle function (i.e., limited physical performance) are all observed, sarcopenia is classified as severe [3]. Muscle strength can be assessed using a Jamar dynamometer, with cutoff values for grip strength defined as < 27 kg for men and < 16 kg for women. Muscle mass can be evaluated using magnetic resonance imaging (MRI), computed tomography (CT), dual-energy X-ray absorptiometry (DXA), or bioelectrical impedance analysis (BIA). Physical performance can be measured using tests such as the Timed Up and Go, Short Physical Performance Battery, or gait speed assessment [3].

EWGSOP2 recommends "Strength, Assistance with walking, Rise from a chair, Climb stairs, and Falls" (SARC-F), a brief five-item questionnaire, as a feasible and rapid screening tool for sarcopenia in clinical settings [3]. Current evidence strongly supports proper nutrition, including adequate protein intake, and exercise training as the most effective strategies for the prevention and management of sarcopenia. Importantly, to date, no medication has received regulatory approval specifically for the treatment of sarcopenia [14].

Sarcopenia was first recognized as a clinical syndrome in 2016 when it was assigned an International Classification of Disease Clinical Modification code (ICD-10-CM). The condition of sarcopenia has gained increased attention in recent years due to its’ association with adverse health outcomes, including frailty, disability, hospitalization, reduced quality of life, and increased mortality in older adults [9, 10, 24, 25]. The development of sarcopenia is influenced by multiple factors, including age-related hormonal changes, inactivity, malnutrition, and chronic illnesses. Like many other diseases, sarcopenia is asymptomatic in initial stages [28]. The condition is not easily identified, therefore remains underdiagnosed and undertreated [11], as its symptoms can be subtle and easily attributed to the aging process. In addition, it is important to note that sarcopenia is distinct from frailty. Sarcopenia refers specifically to the progressive loss of muscle mass, strength, and function, whereas frailty represents a broader clinical syndrome characterized by the presence of three or more of the following criteria: unintentional weight loss (≥ 10 lbs in the past year), self‑reported exhaustion, reduced grip strength, slow walking speed, and low levels of physical activity. Sarcopenia is considered an antecedent and biological substrate of frailty. Recognizing this distinction underscores the need to screen for sarcopenia in older adults as a preventive measure against frailty [2, 8].

As the global demographic shifts towards an increasingly older population, the prevalence of sarcopenia is expected to rise [28], leading to important implications for health care systems and patient outcomes. According to survey data from the United States (U.S.), sarcopenia is estimated to increase hospitalization costs by 34% for older adults and contribute to 1.5% of total health‐care costs in the U.S. [19]. Due to high healthcare costs associated with hospitalization, outpatient clinic visits, and home healthcare expenditure, sarcopenia is a public health burden when not prevented, identified, and managed properly [19, 28].

On the frontline of healthcare, nurses play a pivotal role in the early identification, management, and education of patients at risk for sarcopenia [16]. To effectively manage sarcopenia, nurses must be equipped with the knowledge to assess muscle mass and strength, recognize risk factors, and implement appropriate interventions, such as nutritional support and exercise programs [3, 6]. However, as with any disease management, nurses' ability to identify sarcopenia and implement appropriate interventions is largely determined by their level of knowledge and understanding of the condition. Yet, nurses lack standard guidelines and advanced education regarding identification and management of sarcopenia in the adult and older adult populations [27].

The extent of nurses' knowledge about sarcopenia can vary widely, influenced by factors such as education, clinical experience, and access to continuing education resources [11, 12, 21]. Understanding the current knowledge, perception, and practice of nurses towards sarcopenia is essential to develop targeted education and training programs that can enhance nurses’ competencies in this area of research. Despite the clinical significance of sarcopenia, there are limited research studies about nurses’ knowledge, perceptions, and practice regarding sarcopenia worldwide, particularly in the U.S. During a literature search, only two studies were found, conducted in China and Thailand, that demonstrated nurses have a general lack of knowledge on sarcopenia [11, 12], while other studies have revealed low awareness and limited knowledge of sarcopenia among healthcare professionals, which may hinder its timely diagnosis and effective management [19, 28, 29]. The lack of studies amongst nurses in the U.S. underscores the urgent need to evaluate and enhance nurses’ knowledge on sarcopenia to improve patient outcomes, decrease the burden of health care costs associated with sarcopenia, and promote healthy aging [19, 23]). The purpose of this study is to describe the nurses’ knowledge, perceptions, and practice towards sarcopenia.

## Methods

### Design and sample

A descriptive research design was used in this study. The study was conducted in Wisconsin State (WI). Eligibility was determined through pre-survey screening questions that aligned with the predefined inclusion criteria: 1) being a nurse affiliated with the American Nurses Association (ANA), 2) being a Registered Nurse (RN) working in various healthcare settings in WI, including hospitals, clinics, long-term care facilities, and community health centers; 3) having direct patient care responsibilities or involvement in the care of older adults aged 65 years and above. Student members of the ANA were excluded from the study. Additionally, nurses who primarily work in administrative or managerial roles without direct patient care responsibilities and those who are currently undergoing specialized training or education programs that could influence their knowledge, perceptions, or practice related to sarcopenia were also excluded.

A recruitment email was sent randomly to 14,000 ANA nurse members with an invitation to participate in the study anonymously, with two email prompts each week. A Qualtrics survey link was provided in the email, which in turn brought the participant to the research consent form. Upon clicking consent, the survey continued only with respondents who passed the initial eligibility criteria, identified through the pre-survey questions. All collected data were housed in Qualtrics protected by password and two-step verification.

### Measurements

To assess nurses' self-perceived knowledge, perceptions and practice towards sarcopenia in older adults, a structured questionnaire was developed, adapted, and modified from the elements of previous research studies [13, 28]. The questionnaire was organized into the following categories: demographic variables, nurses’ self-perceived knowledge of sarcopenia, diagnostic strategies, treatment and management of sarcopenia, and enablers and barriers to sarcopenia diagnosis and treatment. The questions assessed nurses’ self-perceived knowledge and perceived responsibility for managing the condition, and familiarity with current management guidelines. The questionnaire also included items evaluating knowledge of age-related changes in muscle mass and strength, including the understanding that both peak in early adulthood and gradually decline thereafter. Additional questions addressed diagnostic criteria, such as established cutoff points for low handgrip strength, and awareness of commonly used assessment tools for evaluating muscle mass, including MRI, CT, and BIA. To ensure clarity, readability, and validity of the questionnaire, five nursing faculty and reviewed the survey. Overall, the structured questionnaire served as a measurement tool for capturing essential demographic and knowledge-related data among nurses regarding sarcopenia.

### Ethical considerations

The study was considered exempt by the institutional review board of the University of Wisconsin—Milwaukee. The study was anonymous, and all participants agreed to participate by signing an informed consent form.

### Data analysis

The Statistical Package for Social Sciences (SPSS Version 29) was used for data analysis. Descriptive statistics were used to summarize participants’ demographic characteristics, as well as their knowledge, perceptions, and practice related to sarcopenia. All categorical variables (e.g., age, gender, professional role, participation in sarcopenia-related training) were summarized using frequencies and percentages. Respondents with missing data (n = 6) were excluded from the analysis.

## Results

A total of 109 electronic questionnaires were successfully obtained, with a participation rate of approximately 1%. The sample characteristics are depicted in Table 1. The median age range (years) of the nurses sampled was between 40–49 years old. From the data collected, most of the nurses were women (89.9%). Around half of the nurses surveyed worked in a hospital setting (45.9%), and 10.1% in outpatient/inpatient clinics, and only 8.3% in nursing home/assisted living. All participating nurses had at least a bachelor’s degree. Nearly half of the nurses (44%) had more than 20 years of nursing experience, and one-third (33%) had over 20 years of experience working with older adults.

**Table 1:** Sample Characteristics (*n* = 109)

Age (years)	n (%)
18–29	21 (19.3)
30–39	22 (20.2)
40–49	25 (22.9)
50–59	20 (18.3)
60 and above	21 (19.3)
**Gender**	**n (%)**
Man	10 (9.2)
Woman	98 (89.9)
Gender not indicated	1 (0.9)
**Level of practice**	**n (%)**
Registered nurse	55 (50.5)
Registered nurse currently enrolled in graduate program	27 (24.8)
Advance practice registered nurse	6 (5.5)
Advance practice registered nurse enrolled in graduate program	21 (19.3)
**Practice setting**	**n (%)**
Hospital	50 (45.9)
Primary care	6 (5.5)
Community health	2 (1.8)
Nursing home/assisted living	9 (8.3)
Outpatient/Inpatient clinic	11 (10.1)
Others	31 (28.4)
**Geographical setting of workplace**	**n (%)**
Metropolitan	34 (31.2)
Urban	49 (45.0)
Regional	12 (11.0)
Rural	14 (12.8)
**Highest level of education**	**n (%)**
Bachelor’s degree	45 (41.2)
Postgraduate certificate of diploma	40 (36.7)
Master’s degree	13 (11.9)
Doctorate	11 (10.1)
**Years worked as a nurse**	**n (%)**
Less than 1 year	8 (7.3)
1 to 5	19 (17.4)
6 to 10	16 (14.7)
11 to 20	18 (16.5)
More than 20	48 (44.0)
**Years working with older adult patients**	**n (%)**
Less than 1 year	16 (14.7)
1 to 5	23 (21.1)
6 to 10	16 (14.7)
11 to 20	18 (16.5)
More than 20	36 (33.0)

### Knowledge of sarcopenia

Table 2 represents the current level of knowledge regarding sarcopenia amongst nurses. The results indicate that half of the nurses generally had limited knowledge of sarcopenia (51.4%) and lacked understanding that sarcopenia is a preventable condition (54.1%). In addition, 15.6% of the nurses explicitly disagreed that sarcopenia can be prevented.

**Table 2:** Knowledge of Sarcopenia (*n* = 109)

Describe your knowledge of sarcopenia	n (%)
Poor	56 (51.4)
Below average	21 (19.3)
Average	29 (26.6)
Good	3 (2.8)
**Sarcopenia can be prevented**	**n (%)**
Agree	33 (30.3)
Disagree	17 (15.6)
I don’t know	59 (54.1)
**Have you received any sarcopenia-related education in the last 12 months?**	**n (%)**
Yes	6 (5.5)
No	103 (94.5)
**What type of education received in the last 12 months?**	**n (%)**
Seminar/Workshop	1 (0.92)
Online training	1 (0.92)
Others	4 (3.67)
None	103 (94.5)
**What are the reasons for the lack of knowledge of sarcopenia? (multiple responses)**	**n (%)**
Busy curriculum	25 (17.7)
Lack of interest	10 (7.1)
No professional training	86 (61.0)
Others	20 (14.2)
**Obesity is a risk factor for sarcopenia**	**n (%)**
Agree	49 (45.0)
Disagree	3 (2.8)
I don’t know	57 (52.3)
**Muscle mass and strength peak in early adulthood, followed by a gradual decline at the age of**	**n (%)**
25	4 (3.7)
30	20 (18.3)
35	14 (12.8)
40	19 (17.4)
45	14 (12.8)
I don’t know	38 (34.9)
**According to the European Working Group on Sarcopenia in Older People (EWGSOP2), low handgrip strength is defined as less than**	**n (%)**
17 kg for male and 6 kg for female	6 (5.5)
37 kg for male and 26 kg for female	4 (3.7)
27 kg for male and 16 kg for female	5 (4.6)
25 kg for male and 15 kg for female	9 (8.3)
I don’t know	85 (78.0)
**Do you want to receive sarcopenia-related education?**	**n (%)**
Yes	76 (69.7)
No	33 (30.3)

Most nurses reported that they had not received education regarding sarcopenia in the last 12 months (94.5%), and only 5.5% reported that they had received education, such as a workshop or online training. The most frequently selected reason for the lack of knowledge of sarcopenia was the absence of professional training (61.0%), followed by a busy curriculum among nurses as students continuing their professional education (17.7%), other unspecified reasons (14.2%), and the lack of knowledge to a general lack of interest in the topic (7.1%).

Notably, more than half of the nurses (52.3% selected “I don’t know” and 2.8% selected “disagree”) were unable to identify obesity as a risk factor for developing sarcopenia. Around 43.0% of nurses overestimated the age at which muscle mass and muscle strength start to decline, and 34.9% of nurses did not know the age at which muscle mass and strength peak in early adulthood. Only 18.3% correctly identified the peak age as 30 years.

Knowledge regarding the sex-specific cut-off values for low handgrip strength was also limited. Only 4.6% of nurses answered correctly, while 78.0% reported not knowing the correct handgrip strength cut-off points. Interestingly, around 70.0% of the nurses expressed interest in receiving sarcopenia-related education.

### Diagnostic strategy, treatment, and management of sarcopenia

Table 3 shows responses regarding diagnostic strategies that can be used in clinical practice to identify sarcopenia. Most of the nurses (96.3%) reported not using an objective algorithm, protocol, or flowchart to evaluate muscle mass, muscle strength, or function among older adults. Furthermore, most nurses (95.4%) did not know how sarcopenia is diagnosed, and more than half (68.8%) were unable to identify some of the most commonly used diagnostic criteria used to diagnose sarcopenia worldwide.

**Table 3:** Diagnostic Strategy (*n* = 109)

Do you use any algorithm/protocol/flowchart to evaluate the muscle mass, muscle strength or function?	n (%)
Yes	4 (3.7)
No	105 (96.3)
**Do you know how sarcopenia can be diagnosed?**	**n (%)**
Yes	5 (4.6)
No	104 (95.4)
**What criteria can be applied to diagnose sarcopenia? (Select the most applied criteria)**	**n (%)**
European Working Group on Sarcopenia in Older Persons	4 (3.7)
International Working Group on Sarcopenia	1 (0.9)
Foundation for the National Institute of Health	1 (0.9)
Appendicular lean mass index	2 (1.8)
Frailty Criteria	26 (23.8)
I don’t know	75 (68.8)
**What tool can be used to diagnose sarcopenia based on muscle mass? (Select the most commonly used tool)**	**n (%)**
Calf circumference	2 (1.8)
Skinfold	2 (1.8)
DXA	3 (2.8)
BIA	3 (2.8)
MRI	1 (0.9)
CT	3 (2.8)
None	95 (87.2)

*DAX* Dual-energy X-ray absorptiometry, *BIA* Bioelectrical Impendence Analyzer, *CT* Computed tomography, *MRI* Magnetic resonance imaging

Around 23.8% of nurses selected frailty criteria as diagnostic criteria for diagnosing sarcopenia, indicating confusion between the concepts of sarcopenia and frailty. Moreover, most nurses (87.2%) were unaware of the tools that can be used to assess body composition, including muscle mass, when diagnosing sarcopenia. Only a small proportion of nurses correctly identified various diagnostic tools used to assess body composition. Specifically, 1.8% of nurses recognized calf circumference and skinfold measurements, while 2.8% correctly identified DXA, 2.8% recognized BIA, 2.8% recognized CT imaging as appropriate tools, and only 0.9% recognized MRI as diagnostic tools for assessing body composition.

Table 4 summarizes nurses’ responses to questions on the treatment and management of sarcopenia. When asked about the importance of sarcopenia in the overall management of older adults, 22.0% of nurses considered it extremely important, 30.3% rated it as very, and 31.2% as somewhat important. A smaller proportion viewed it as minimally important (12.8%) or not important (3.7%). Regarding treatment strategies, resistance exercises were the most frequently selected intervention (18.5%), followed by nutritional intervention (16.6%), protein supplementation (15.4%), balance exercises (14.7%), aerobic exercises (9.4%), Vitamin D (10.0%), and pharmacological interventions (3.7%). Around 11.6% of the nurses responded: “I don’t know”, demonstrating a lack of knowledge regarding treatment strategies for sarcopenia.

**Table 4:** Treatment and Management of Sarcopenia (*n* = 109)

How important is sarcopenia in the overall management of older adults?	n (%)
Extremely important	24 (22.0)
Very important	33 (30.3)
Somewhat important	34 (31.2)
Minimally important	14 (12.8)
Not important	4 (3.7)
**What should sarcopenia be treated with? (multiple responses)**	**n (%)**
Aerobic exercises	30 (9.4)
Nutritional intervention	53 (16.6)
Protein supplements	49 (15.4)
Resistance exercises	59 (18.5)
Vitamin D	32 (10.0)
Balance exercises	47 (14.7)
Pharmacological intervention	12 (3.7)
I don’t know	37 (11.6)

### Perception of barriers and enablers in sarcopenia diagnosis and treatment

Table 5 summarizes nurses’ perspectives on the factors, both enablers and barriers, that influence the diagnosis and treatment of sarcopenia. The most frequently selected response was “I don’t know” (41.0%), reflecting a considerable degree of uncertainty or lack of awareness regarding existing enablers. Among the identified enablers, the most reported was access to training on appropriate and available screening processes (14.9%), followed by the implementation of screening protocols (13.3%), the presence of key performance indicators to meet for screening (12.2%), recognition of sarcopenia identification as a priority within the healthcare setting (9.6%), and clarity regarding which professional role is responsible for screening (9.0%).

**Table 5:** Enablers and barriers in sarcopenia diagnosis and treatment (*n* = 109)

What do you consider the enablers to the identification of sarcopenia in older adults?	n (%)
Access to training on appropriate screening processes available	28 (14.9)
Protocols are implemented to support the processes for screening	25 (13.3)
There are key performance indicators to meet for screening	23 (12.2)
It is clear whose role screening is in your organization	17 (9.0)
Identifying the condition is a priority for my health care setting	18 (9.6)
I don’t know	77 (41.0)
**What are the barriers to identification of sarcopenia in clinical settings? (multiple responses)**	**n (%)**
Lack of awareness among other healthcare professionals including nurses	30 (14.4)
Acquisition of a device to measure muscle mass	13 (6.2)
I am not trained to measure muscle mass	59 (28.2)
Acquisition of handgrip strength device	13 (6.2)
I do not have the skill in measuring handgrip strength	21 (10)
No space for walking test	3 (1.4)
Time constraints to perform the diagnostic tests	12 (5.7)
No funding source specific for sarcopenia	11 (5.3)
Other	16 (7.7)
**Is there a protocol for diagnosing sarcopenia at your workplace?**	**n (%)**
Yes	1 (0.9)
No	32 (29.4)
I don’t know	76 (69.7)
**Who do you think is responsible for identifying sarcopenia in patients at your work setting? (multiple responses)**	**n (%)**
Physician	51 (21.9)
Physician of Physiology	21 (9.0)
Exercise Physiologist	23 (9.9)
Nurse	29 (12.4)
Nursing Assistant/Dentition Assistant/Allied Health Assistant	7 (3.0)
Medical team	30 (12.9)
Physiotherapist/Physical therapist/Doctor of physical therapy	40 (17.2)
Others	9 (3.9)
I don’t know	23 (9.9)

When asked about barriers experienced during sarcopenia diagnosis, the most frequently selected response was a lack of training to measure muscle mass (28.2%), followed by lack of awareness (14.4%), lack of skills in measuring handgrip strength (10.0%), limited acquisition of devices to measure muscle mass (6.2%) and handgrip strength (6.2%), time constraints (5.7%), lack of sarcopenia-specific funding (5.3%), and lack of space for conducting walking tests (1.4%).

Furthermore, 69.7% of the nurses demonstrated a lack of knowledge regarding the presence of a protocol for diagnosing sarcopenia in their workplace, while 29.4% reported no existing protocol and only 0.9% indicated that such a protocol was in place. Regarding the responsibility for identifying sarcopenia, the most frequently selected response was physicians (21.9%), followed by physiotherapist/physical therapist/doctor of physical therapy (17.2%) and the broader medical team (12.9%), nurses (12.4%), exercise physiologists (9.9%), physiologists (9.0%), nursing or allied health assistants (3.0%). Around 9.9% of the nurses demonstrated a lack of knowledge regarding who is responsible for identifying sarcopenia among patients in their health service setting.

## Discussion

To the best of our knowledge, this study was the first to assess the knowledge, perceptions, and practice of sarcopenia among registered nurses within the U.S. The findings of this study indicate an important gap in knowledge regarding sarcopenia amongst nurses. These findings also raise concerns about nurses’ awareness and understanding of sarcopenia as a prominent health condition that affects older adults. The results demonstrate that more than half of nurses reported a limited knowledge of sarcopenia and limitations in prevention, assessment, management strategies, and diagnostic criteria for sarcopenia. These results are consistent with studies that reported a universal lack of knowledge of sarcopenia, its associated sequelae, and its prevention and management amongst health care professionals [10, 12, 19, 23, 28]. It is important to note that limited understanding of sarcopenia is not unique to nurses; similar knowledge gaps have been documented among physicians and other healthcare professionals [10, 27]. This challenge is compounded by the existence of multiple definitions of sarcopenia and the absence of universally accepted guidelines or consensus on diagnostic criteria, assessment tools, and cutoff points [7]. Besides the EWGSOP, several groups from different countries have proposed their own definitions and diagnostic criteria,however, the EWGSOP remains one of the most commonly used and widely accepted frameworks. Such variability contributes to inconsistent recognition, assessment, and management of the condition in clinical practice. Acknowledging this broader complexity highlights the need for clearer, standardized frameworks to support healthcare providers in accurately identifying and addressing sarcopenia across care settings [7, 10, 27].

Most nurses were unaware that obesity is a risk factor for sarcopenia. In addition to low muscle mass and strength, increased adiposity can coexist with sarcopenia, a condition known as sarcopenic obesity [17]. This phenotype is associated with poorer functional outcomes and a higher risk of morbidity compared to sarcopenia or obesity alone. Recognizing sarcopenic obesity is important for early identification and for tailoring interventions in older adults, as it may influence both diagnostic approaches and management strategies [17].

The lack of knowledge and awareness of sarcopenia is of critical concern, given its increasing prevalence among older adults and significant implications for patient care. Muscle weakness, a central feature of sarcopenia, was observed in approximately 5% of U.S. adults aged 60 and older, according to national survey data from 2011–2012 [15], while more recent studies report that the prevalence of sarcopenia in the United States ranges from 9 to 18% [26]. Without adequate training and awareness, nurses may struggle to identify at-risk patients, leading to delayed interventions, negative health outcomes, and increased healthcare expenditure [4, 21].

In this current study, lack of professional training and busy curriculum were the primary reasons for limited knowledge regarding sarcopenia among nurses. Most nurses also reported not receiving any sarcopenia-related education in the past year. A recent study reported that most nurses had neither engaged in gerontology training nor received education on sarcopenia during or after graduation, which may explain their limited knowledge of the condition [21]. A critical barrier to nursing training is the shortage of personnel, which results in excessive workloads and limited time for professional development [11, 12]. Consequently, training opportunities in specialized areas such as sarcopenia are often neglected, contributing to persistent gaps in knowledge and practice. Concurrently, other studies have shown the impact of incorporating sarcopenia into continuing education for broader healthcare professionals [10, 12, 13, 27, 28]. Encouragingly, around two-thirds of the nurses expressed a desire to receive sarcopenia-related education. These findings highlight the need for educational interventions focused on sarcopenia to increase the nurses' knowledge and enhance their competencies in effectively screening and managing this condition.

Our results also demonstrate that nurses lacked knowledge about how to identify sarcopenia and were unaware of the tools that can be used to assess body composition, including muscle mass. Nurses also reported not using any algorithm, protocol, or flowchart to evaluate muscle mass, strength, or physical performance among older adults. These findings are consistent with previous studies [10, 19, 28] and highlight the need for early documentation of key indicators of sarcopenia and the integration of standardized assessment tools into the medical health record to support timely documentation, identification, and intervention.

In addition, while most nurses did not know the criteria that can be applied to diagnose sarcopenia, the results revealed confusion between frailty and sarcopenia. A considerable number of nurses in the study sample were confused about the definition of sarcopenia, interrelating the sarcopenia condition with the frailty phenotype in older adults. Several participants mistakenly indicated that frailty criteria, such as unintentional weight loss, exhaustion, and reduced physical activity [8], can be applied to diagnose sarcopenia, instead of focusing on the key diagnostic indicator of muscle strength. This suggests a need for clear clinical guidance and education to distinguish between these two geriatric conditions. Sarcopenia is a major precursor to frailty,therefore, recognizing and understanding sarcopenia is essential for preventing the onset of frailty [18]. Moreover, most nurses were unaware of the tools for assessing body composition, including muscle mass. Only a small proportion identified assessment methods such as DXA, BIA, MRI, CT, and simple anthropometric measures such as calf circumference and skinfold thickness. It is crucial to note that anthropometric measures, such as calf circumference and skinfold, are sometimes used to reflect nutritional status in older adults and have been employed in some studies as a proxy for assessing muscle mass in settings where more precise diagnostic methods are unavailable. However, these measures are not reliable indicators of muscle mass, as noted by the EWGSOP2 [3].

Although many nurses recognize the importance of sarcopenia in the overall management of older adults, there were common perceived barriers to effective screening and treatment, including the lack of awareness, skills, training, and tools to assess sarcopenia-related key indicators, including muscle mass and strength. These barriers are multifaceted and align with findings from previous research [10, 11, 19, 21, 28]. The lack of awareness, skills, and training among healthcare professionals, including nurses, suggests a systematic knowledge deficit within the healthcare system. This issue is further exacerbated by the absence of standardized protocols for diagnosing sarcopenia. These challenges underscore the urgent need for accessible, standardized diagnostic resources to support prompt identification and management of sarcopenia in clinical settings. Equipping nurses with the necessary knowledge, competencies, and resources will enhance their ability to effectively identify, assess, and manage sarcopenia, ultimately improving patient outcomes [4, 21].

### Implications

To thrive in the field of nursing, one must discern factors that can enhance or hinder their professional development in the care of adults and older adults. The results of this study indicate the need for education programs regarding sarcopenia and healthcare facilities to incorporate targeted training on sarcopenia prevention and treatment. Such training would enable nurses to identify and manage sarcopenia more effectively, thereby reducing adverse outcomes and incidence among older adults. Programs should cover the definition, risk factors, identification methods, and prevention and treatment strategies, emphasizing self‑management behaviors like physical activity and healthy dietary practices to enhance muscle mass, strength, and performance [1, 11, 21, 22], [25].

Although education can be the first step to increase knowledge and raise awareness, more multidisciplinary and systematic strategies are needed for successful implementation. For example, improving role clarity regarding sarcopenia identification and management among the multidisciplinary team and developing a nursing practice guideline for early identification and preventing sarcopenia can be beneficial [11, 20, 21]. Furthermore, given the crucial public health consequences of sarcopenia, government and healthcare institutions should allocate funding and other resources to support diagnostic tools, workforce development, motivation and rewards, as well as foster collaboration efforts among healthcare providers including nurses [19, 27, 28] all of which have the potential to improve nurse knowledge, attitudes and practice toward sarcopenia, enhance patient outcomes, and decrease the incidence of sarcopenia.

### Limitations

This study has several limitations. First, the overall response rate was low, raising concerns about potential self-selection bias and limiting the representativeness of the findings. Second, excluded cases provided very minimal responses, which limited our ability to compare them with respondents who completed the full survey. Third, although steps were taken to design and order survey questions in a way that reduced learning effects, it remains possible that participants adjusted their responses as they progressed through the questionnaire. Social desirability bias may also have led some respondents to overestimate their knowledge or competencies related to sarcopenia [5]. Fourth, because the survey relied on self-reported data, responses may not fully reflect actual knowledge or practice behaviors. In addition, nurses who were not affiliated with the ANA, as well as those currently engaged in specialized training or educational programs that might affect their knowledge or practice regarding sarcopenia, were excluded, which may limit the generalizability of the results to the broader nursing population. Finally, the questionnaire was adopted for this study and has not been formally validated,as such, its ability to fully capture the construct of perceived knowledge may be limited. Although content clarity and consistency were carefully considered during development, the findings should be interpreted with caution considering these limitations.

## Conclusion

While nurses play a crucial role in the early identification, management, and education of patients at risk for sarcopenia, this study demonstrated that they lacked adequate knowledge about sarcopenia and were unfamiliar with appropriate methods for its early identification and treatment. Although nurses had not received formal training on sarcopenia, they expressed strong interest in obtaining such training. These findings highlight the need for enhanced education and training, clinical guidelines, and standardized assessment processes to support timely identification and effective management of sarcopenia.

## Data Availability

The datasets used and/or analyzed during the current study are available from the corresponding author on reasonable request.

## Abbreviations

EWGSOP2: The European Working Group on Sarcopenia in Older People
ICD-10-CM: International Classification of Disease Clinical Modification code)
U.S.: United States
ANA: American Nurses Association
IRB: Institutional Review Board
SPSS: Statistical Package for the Social Sciences
DXA: Dual-energy X-ray Absorptiometry
BIA: Bioelectrical Impedance Analysis
CT: Computed Tomography
MRI: Magnetic Resonance Imaging
